# Supplementary material for: Health professionals’ technology readiness on the acceptance of teleradiology in the Amhara regional state public hospitals, northwest Ethiopia: Using technology readiness acceptance model (TRAM)
Source: PLoS One. 2024 Mar 28;19(3):e0301021. doi: 10.1371/journal.pone.0301021 (PMC10977896; doi:10.1371/journal.pone.0301021)
Supplement: Attachment — Submitted filename: Review on TRAM.docx [file pone.0301021.s010.docx]

The paper is well articulated and it is timely. Conducting such types of studies “Health professionals’ technology readiness on the acceptance of teleradiology” will enhance the telemedicine penetration and improve the health service coverage by increasing health access of the individuals. Over all the manuscript is well written, but I have some questions and comments that have to be addressed by the authors to improve the quality of the paper.

1. It would be better to add some information on the Telemedicine/Teleradiology practice in Amhara region of Ethiopia, even pilot implementations.
2. Some of the questions in the model assessment you had asked Perceived Ease of Use. Do you think it is easy to answer this question without having a single exposure on Telemedicine before?
3. For the qualitative part you only asked eight key informants from different sectors, do you think it is sufficient? Did the data saturate at the 8^th^ key informant? Make it clear.
4. In reporting the qualitative finding rather than saying he/she it is better to describe the respondent keeping them anonymous.
