# Supplementary material for: Health professionals’ technology readiness on the acceptance of teleradiology in the Amhara regional state public hospitals, northwest Ethiopia: Using technology readiness acceptance model (TRAM)
Source: PLoS One. 2024 Mar 28;19(3):e0301021. doi: 10.1371/journal.pone.0301021 (PMC10977896; doi:10.1371/journal.pone.0301021)
Supplement: Attachment — Submitted filename: Response to Reviewers.docx [file pone.0301021.s011.docx]

Rebuttal letter *(point-by-point response)* to the academic editor and reviewer

We appreciate you the academic editor and the reviewer for your precious time in reviewing our paper and providing valuable and scientific comments and suggestions. It was your valuable and insightful comments, questions and suggestions that led to possible improvements in the current manuscript version. The authors have carefully considered the comments, questions and suggestions and tried our best to address every one of them.

| No | **Editors and Reviewer’s Comments** | |
| --- | --- | --- |
| I. | **Editor’s comments** | **Authors’ response** |
|  | A rebuttal letter that responds to each point raised by the academic editor and reviewer(s). You should upload this letter as a separate file labeled 'Response to Reviewers'. | Thank you for your suggestion!  We uploaded our point by point response (rebuttal letter) as separate word file named as 'Response to Reviewers'. |
|  | A marked-up copy of your manuscript that highlights changes made to the original version. You should upload this as a separate file labeled 'Revised Manuscript with Track Changes'. | Thank you for your suggestion!  We highlighted the modification using track changes and we uploaded a marked-up copy of our manuscript as separate word file named as 'Revised Manuscript with Track Changes'. |
|  | An unmarked version of your revised paper without tracked changes. You should upload this as a separate file labeled 'Manuscript'. | Thank you for your suggestion!  We uploaded a revised cleaned separate manuscript word file named as 'Manuscript'. |
|  | 1) Please ensure that your manuscript meets PLOS ONE's style requirements, including those for file naming. | Thank you for your valuable suggestion!  We tried to follow the journal submission guidelines. |
|  | 2a). Please provide an amended statement that declares *all* the funding or sources of support (whether external or internal to your organization) received during this study, as detailed online in our guide for authors at <http://journals.plos.org/plosone/s/submit-now> | Thank you editor for your comment!  We did modification on the funding section. [Funding statement section: Page 31; line numbers 642-644]. |
|  | 2b). Please also include the statement “There was no additional external funding received for this study.” in your updated Funding Statement. | Thank you for the suggestion!  We did the amendment accordingly.  [Funding statement section: Page 31; line number 644]. |
|  | 2c). Please include your amended Funding Statement within your cover letter | Thank you editor for your relevant comment.  We considered he comment and included it accordingly. [Cover letter]. |
|  | In your revised cover letter, please address the following prompts:  3a) If there are ethical or legal restrictions on sharing a de-identified data set, please explain them in detail (e.g., data contain potentially sensitive information, data are owned by a third-party organization, etc.) and who has imposed them (e.g., an ethics committee). Please also provide contact information for a data access committee, ethics committee, or other institutional body to which data requests may be sent. | Thank you very much dear editor!  There is no legal restrictions, we uploaded all the data set relevant to this manuscript. |
|  | 3 b) If there are no restrictions, please upload the minimal anonymized data set necessary to replicate your study findings as either Supporting Information files or to a stable, public repository and provide us with the relevant URLs, DOIs, or accession numbers. | Thank you, editor, for your question!  We uploaded the all the relevant data sets: [Supporting information: Page 32 line numbers 664 to 667]. |
|  | 4. We note that the grant information you provided in the ‘Funding Information’ and ‘Financial Disclosure’ sections do not match.  When you resubmit, please ensure that you provide the correct grant numbers for the awards you received for your study in the ‘Funding Information’ section. | Thank you for the relevant comment.  We did amendment for the mismatch by correcting the online financial disclosure section grant number (Ref. No: 05/2886/2013). |
|  | 5) Please remove any funding-related text from the manuscript and let us know how you would like to update your Funding Statement.  Please include your amended statements within your cover letter; we will change the online submission form on your behalf. | Thank you for the suggestion!  We did the amendment accordingly.  [Acknowledgment section: Page 31; line numbers 629].  We also amended the cover letter. [Cover letter] |
|  | 6) Please include captions for your Supporting Information files at the end of your manuscript, and update any in-text citations to match accordingly | Thank you for the suggestion!  We did the amendment accordingly.  [Supporting information: Page 32; line number 663]. |
|  | **Additional Editor Comments** |  |
|  | The paper has merit. However, before recommending the publication, I suggest addressing the following issues. | Thank you very much for your constructive comments and suggestions! |
|  | 1) The introduction lacks proper identification of research questions. Even the authors did not provide clear logic as to why this study is important. | Thank you editor for your insightful comment. We did extensive revision according to your suggestion. [Background section: Pages 3 to 6] |
|  | 2. The logic behind constructing the hypothesis lacks. - Why qualitative component needed after quantitative evidence?  - What is the main motivation for studying the qualitative component? | Thank you editor for your valuable comment.   - We did revision according to your suggestion on the theoretical model and hypothesis development sections. [Background section: Pages 6 to 9] - We added a qualitative component to our study to address organizational related barriers contributing for the technology adoption. [Background section: Pages 3 |
|  | Does the discussion section provide sufficient information on the research questions? (though research questions have lacked). | Thank you editor for the very important questions!   - We did extensive revision according to your suggestion. [Discussion section: Page Numbers 24 - 29] - We added research questions. [Background section: Pages 4; Line numbers 79 - 80] |
|  | There is a lack of both theoretical and practical contributions.  - For example, how does this study contribute to the scholarly world or extend the scope of existing literature?  - How does this study contribute to the health professionals in a specific manner?  - How would this study be beneficial for Ethiopian public and private healthcare systems/industry? | Thank you editor for constructive comment!   - We have tried to address accordingly. [Background section: Page No 4; Line Numbers 81-85] |
| **II** | **Reviewer’s comments** | |
|  | 1. It would be better to add some information on the Telemedicine/Teleradiology practice in Amhara region of Ethiopia, even pilot implementations. | Thank you reviewer for your constructive comment.  We conducted a thorough search for documents on teleradiology implementation, but unfortunately, we were unable to find any information regarding its status in Ethiopia's public health facilities, including the Amhara region. However, we did come across a digital health blueprint plan document that outlines the implementation of health technologies by the Ministry of Health. (<https://ethiopiadup.jsi.com/resource/ethiopia-digital-health-blueprint/>)  [Introduction section: Page 3; Line numbers 62-65] |
|  | 2. Some of the questions in the model assessment you had asked Perceived Ease of Use. Do you think it is easy to answer this question without having a single exposure on Telemedicine before? | Thank you for the important question.  Of course, asking perceived ease use questions regarding telemedicine for those people who didn’t have pervious exposure is challenging. However, in this study we included only professionals who were familiar with telemedicine concept at least in theory graduate and/or postgraduate classes. Hence, considering their prior theoretical exposure, we included participants from imaging, medicine, internal medicine, medical, dermatology, oncology, pathology, pediatrics, gynecology departments. |
|  | 3. For the qualitative part you only asked eight key informants from different sectors, do you think that it is sufficient? Did the data saturate at the 8^th^ key informant? Make it clear. | Thank you for the valuable question.  Yes! We contacted only 8 key informants considering point of saturation. Since our intention was to explore organizational barriers affecting teleradiology adoption, questions were more specific and were related to infrastructures, budget, and manpower. As a result, we reached saturation after contacting 8 key informants. |
|  | 4. In reporting the qualitative finding rather than saying he/she it is better to describe the respondent keeping them anonymous | Thank you for the valuable comment.  We did modification accordingly. [Result Section: Page 23; line number 457] |

Thank you!
